# Circulating tumor cells in pulmonary vein and peripheral arterial provide a metric for PD-L1 diagnosis and prognosis of patients with non-small cell lung cancer

**DOI:** 10.1371/journal.pone.0220306

**Published:** 2019-07-26

**Authors:** Jingsi Dong, Daxing Zhu, Xiaojun Tang, Dan Lu, Xiaoming Qiu, Bingjie Li, Dan Lin, Lu Li, Jiewei Liu, Qinghua Zhou

**Affiliations:** 1 Department of Lung Cancer Center, Sichuan University West China Hospital, Chengdu, China; 2 Department of Otorhinolaryngology, Head & Neck Surgery, Sichuan University West China Hospital, Chengdu, China; National Cancer Center, JAPAN

## Abstract

**Background:**

Lung cancer is the leading cause of death caused by malignant tumors. PD-L1(programmed cell death protein-1) has shown tremendous achievement in treating NSCLC. We sought to find the relationship between CTCs in the pulmonary vein and postoperative PFS, besides we detected PD-L1 in CTCs.

**Method:**

We enrolled 112 NSCLC patients. CTC tests were performed at four time points (preoperative, pulmonary vein, intraoperative and postoperative) on every NSCLC patient who received surgery. The RNA of PD-L1 was tested by FISH. The levels of the PD-L1 mRNA and protein in tissue samples were detected.

**Results:**

The CTCs in the PV were the highest (*P*< 0.001), and CTCs in the PPA were the lowest (*P*< 0.001). The PFS in the group with PV CTCs≥ 16/5 ml was shorter than that in the group with PV CTCs< 16/5 ml (11.1 months vs 21.2 months, respectively; *P*< 0.001). The PFS in the group with PPA CTCs≥ 3/5 ml was shorter than that in the group with CTCs< 3/5 ml (14.8 months vs 20.7 months, respectively; *P*< 0.001). The CTCs in stage I were lower than those in stage II-IV (*P* = 0.025). No linear relationship was found between the CTCs and tumor size (*P*> 0.05) or LN metastasis (*P*> 0.05). In total, fifty-two (50.5%) patients had positive PD-L1 expression in CTC. In PD-L1-positive CTC patients, the value of PD-L1 tissue expression was higher than that in PD-L1-negative CTC patients (*P* = 0.0153).

**Conclusion:**

CTCs in the pulmonary vein can be an effective prognosis indicator of NSCLC patients.

## Introduction

Lung cancer, known as a public health problem in the world, is the leading cause of death caused by malignant tumors worldwide. According to Cancer Statistics published in CA, the estimated new cases and deaths caused by lung cancer in 2018 are 234,030 and 154,050, respectively [1]. In China, lung cancer is the most common cancer and the leading cause of cancer death [2]. Despite advances in cancer therapy, the overall five-year survival rate for high grade lung cancer remains less than 20 percent [3]. Non-small cell lung cancer accounts for more than 80 percent of lung cancer cases.

In 2011, data from the National Lung Screening Trial (NLST) showed that early screening can promote the development of early detection, early diagnosis and early intervention; Particularly, CT screening could decrease the total death rate by 19% (RR = 0.81; 95% CI: 0.70~0.92). Treatment for lung cancer depends on the cancer's specific cell type, TNM stage, and person's performance status [4]. Patients with stage I and II disease, are usually treated with surgery. Some patients diagnosed with advanced lung cancer can be treated by chemotherapy, targeted therapy and immunotherapy.

Gaining attention, immunotherapy is considered to be a great opportunity for the treatment of advanced lung cancer. In 2002, Dunn et al. [5] proposed the theory of immune editing. In subsequent research, a large number of activators and inhibitors was found to play important roles in the process of immune response regulation [6]. Currently, two immune checkpoints have been approved as paradigm shifts in immunotherapy: cytotoxic T lymphocyte antigen-4 (CTLA-4) and programmed cell death protein-1(PD-1)/programmed cell death ligand 1 (PD-L1). Inhibitors of PD-1 and its ligand PD-L1 are recommended as first-line and second-line therapies for NSCLC because of their superiority in clinical efficacy and quality of life (QOL). Pembrolizumab, a well-known inhibitor of PD-L1, was used in a phase 3 trial published in 2018 and was demonstrated significantly longer overall survival and progression-free survival and a higher response rate than the addition of placebo [7]. Additionally, pembrolizumab in metastatic NSCLC demonstrated a surprisingly effective outcome (Keynote-021) with an ORR of 55% versus 29% with chemotherapy alone (P = 0.0016) [8]. PD-L1 on the tumor cell membrane can be abnormally upregulated and inhibit the activation of T lymphocytes, leading to tumor immune escape [9]. Any inhibition of PD-1 and PD-L1 will enable T cells to regain the ability to identify and kill tumor cells,by blocking immune escape [10]. PD-L1 has shown tremendous achievements in the treatment of NSCLC.

Tumor cells detached from the primary or metastatic tumor sites that escape death from the body's immune system, and survive in the circulatory system are called circulating tumor cells (CTC) [11]. CTCs are considered to be the leading causes and markers for tumor recurrence and metastasis [12]. The study of CTCs in lung cancer is one of the most hot topics in clinical research. Some studies on CTCs based on specific biomarkers, such as EpCAM, have shown that the detection rate and detection quantity are decreased compared with those of other cancers such as breast cancer and hepatocellular carcinoma [13–15]. Moreover, many clinical studies on the CTCs of NSCLC have shown the reliability of CTCs as a prognostic indicator. Wang’s study indicates that CTC detection is mainly related to tumor stage, lymph node metastasis and prognosis, and CTC detection is significantly associated with the shortening of PFS (progression-free survival) and OS (overall survival) in NSCLC [16]. Hofman’s team tracked the content of CTCs in 208 NSCLC patients for two years and found that the patients with more than 50 circulating nonhematopoietic tumor cells showing a shorter total survival period and disease-free survival period [17]. The pulmonary vein is the closest vein to the tumor. Compared with peripheral blood, the number of circulating tumor cells contained in the pulmonary vein is relatively concentrated and the detection rate is higher. CTC detection in the pulmonary vein and PD-L1 detection in CTC have been studied rarely at present.

The aim of this study was to determine the relationship between the CTCs in blood and TNM stages of NSCLC patients and to confirm whether the CTCs can be an effective prognosis indicator of NSCLC patients. Additionally, we aimed to explore whether PD-L1 detection in circulating tumor cells can be an effective substitute for PD-L1 detection in patients without available pathological tissue and to test and verify whether the method of detecting CTC in pulmonary veins is efficacious in the study of early-stage NSCLC patients.

## Materials and methods

### Patients and study design

One hundred twelve non-small cell lung cancer (NSCLC) patients who received surgical resection at the West China Hospital of Sichuan University were included in this study from December 2016 to January 2018. One hundred three patients met the inclusion criteria. All the patients were informed of the procedure and signed informed consent. The study was approved by the medical ethics committee of Sichuan University. Our report adheres to the REMARK criteria [18].

Histopathological staging of the patients included the tumor size, lymph node status, distant metastasis status, and pathological type. The staging method was carried out according to the staging standard of AJCC eighth edition. The study included stage I-III NSCLC patients. It is noteworthy that nine of the patients showing a small amount of implant metastasis in the pleura when receiving surgery and were classified as stage IV (**Table 1**).

**Table 1:** Clinical characteristics of the enrolled Non-small cell lung cancer patients.

Characteristic	No. (Total = 103)
Age(mean)	59.4(years)
Gender	
Female	54
Male	59
Histology	
Squamous	36
Adenocarcinoma	60
Others*	7
Surgical method	
Lobectomy	75
Segmentectomy/Wedge	16
Sleeve lobectomy	9
Pneumonectomy	3
Stage(AJCC 8)	
Stage I	48
Stage II	19
Stage III	27
Stage IV	9
Neoadjuvant Chemotherapy	
Yes	3
No	100
Neoadjuvant Radiotherapy	
Yes	1
No	102
Adjuvant Chemotherapy	
Yes	39
No	64
Adjuvant Radiotherapy	
Yes	22
No	81
Targeted Therapy	
Yes	7
No	96
Immunotherapy	
Yes	2
No	101

Others*: four of adeno-squamous carcinoma; three of large cell lung cancer

Peripheral arterial blood samples (5 ml) for CTC detection were collected at in two hours before surgery, during the operation (during pulmonary surgery), and one hour after surgery. Blood from pulmonary vein (5 ml) samples for CTC detection were collected during the operation. None of the patients in the study died within 30 days after surgery. The follow-up time was for twenty-four months. The overall survival (OS) time was from surgery to death. The progression-free survival (PFS) was the time from surgery to the time of the diagnosis of local recurrence, distant metastasis or death, whichever occurred first (**Table 2**).

**Table 2:** Probe information.

Gene	Sequence (5’- 3’)
EpCAM	TGGTGCTCGTTGATGAGTCA
	AGCCAGCTTTGAGCAAATGA
	AAAGCCCATCATTGTTCTGG
	CTCTCATCGCAGTCAGGATC
	TCCTTGTCTGTTCTTCTGAC
	CTCAGAGCAGGTTATTTCAG
CK8	CGTACCTTGTCTATGAAGGA
	ACTTGGTCTCCAGCATCTTG
	CCTAAGGTTGTTGATGTAGC
	CTGAGGAAGTTGATCTCGTC
	CAGATGTGTCCGAGATCTGG
	TGACCTCAGCAATGATGCTG
CK18	AGAAAGGACAGGACTCAGGC
	GAGTGGTGAAGCTCATGCTG
	TCAGGTCCTCGATGATCTTG
	CAATCTGCAGAACGATGCGG
	AAGTCATCAGCAGCAAGACG
	CTGCAGTCGTGTGATATTGG
CK19	CTGTAGGAAGTCATGGCGAG
	AAGTCATCTGCAGCCAGACG
	CTGTTCCGTCTCAAACTTGG
	TTCTTCTTCAGGTAGGCCAG
	CTCAGCGTACTGATTTCCTC
	GTGAACCAGGCTTCAGCATC
Vimentin	GAGCGAGAGTGGCAGAGGAC
	CTTTGTCGTTGGTTAGCTGG
	CATATTGCTGACGTACGTCA
	GAGCGCCCCTAAGTTTTTAA
	AAGATTGCAGGGTGTTTTCG
	GGCCAATAGTGTCTTGGTAG
Twist	ACAATGACATCTAGGTCTCC
	CTGGTAGAGGAAGTCGATGT
	CAACTGTTCAGACTTCTATC
	CCTCTTGAGAATGCATGCAT
	TTTCAGTGGCTGATTGGCAC
	TTACCATGGGTCCTCAATAA
CD45	TCGCAATTCTTATGCGACTC
	TGTCATGGAGACAGTCATGT
	GTATTTCCAGCTTCAACTTC
	CCATCAATATAGCTGGCATT
	TTGTGCAGCAATGTATTTCC
	TACTTGAACCATCAGGCATC
PD-L1	CTACTGGGAATTTGCATTCA TAGTGCAGCCAGGTCTAATT TCCTCTCCATGCACAAATTG TGTAGCTACTATGCTGAACC GAGAGCTGGTCCTTCAACAG GATCTGAAGTGCAGCATTTC CATCCTGCAATTTCACATCT CTGATCATGCAGCGGTACAC

All of the tumor cells in the CTC blood samples were evaluated for the expression level of PD-L1 by FISH (fluorescence in situ hybridization), as well as the tumor tissues of all NSCLC patients. The expression of PD-L1 in the tumor tissues of all patients was also determined by immunohistochemistry (IHC) at the same time.

### Blood drawn from the pulmonary vein to detect CTC

In this study, all the included patients received conventional thoracotomy. Before surgery, one 10-ml syringe was prepared containing a small amount of heparin sodium saline (less than 0.5 ml). During the surgery, the roots of the pulmonary vein were ligated at the proximal end of the heart, and then 5 ml of blood was extracted at the distal end of the pulmonary vein. After the blood was extracted from the pulmonary vein, it was immediately injected into the syringe containing EDTA. Next, the proximal end of the pulmonary vein was ligated a second time, and the distal end of the pulmonary vein was ligated finally.

### WBC and neutrophil-to-lymphocyte count

In this study, we collected the venous blood of NSCLC patients before surgery and 1 hour after surgery. WBC(White blood cell) and neutrophil-to-lymphocyte ratio were detected by automated assay(Sysmex, Japan, XN2100).

### CTC measurement

In this study, CanPatrol (Surexam Biotech, Guangzhou, China) was used to identify CTCs in lung adenocarcinoma patients, as previously described[19]. Five milliliters of peripheral (or pulmonary venous) blood in an anticoagulated tube with EDTA was drawn from all the participants before each checkpoint. Mononuclear cells (MNCs) were isolated by adding an erythrocyte lysis buffer which consisted of 154 mM NH4Cl, 10 mM KHCO3, and 0.1 mM EDTA in deionized water (all from Sigma, St. Louis, USA). After centrifugation (1500rpm, 5min), the MNCs were resuspended in PBS (Sigma, St. Louis, USA) containing 4% formaldehyde (Sigma, St. Louis, USA) for 5 minutes. The MNCs suspension was transferred to the filtration tube (Surexam Biotech, Guangzhou, China) containing a calibrated membrane with 8-μm diameter pores (Millipore, USA) and pumped with at least 0.08MPa. The CTCs, which are larger than the other MNCs, were ultimately retained on the membrane, while the other MNCs passed through the filter pores.

A multiplex RNA-in situ hybridization (RNA-ISH) assay was conducted in a 24-well plate (Corning, NY, USA) after the membrane with 8-μm diameter pores was removed from the filtration tube to classify and count CTCs. Four epithelial markers (EpCAM and CK8/18/19), two mesenchymal markers, and a leukocyte marker (CD45) were applied to capture and characterize the CTCs. The detail RNA-ISH assay procedure followed the published literature[19]. Briefly, the cells retained on the membrane were treated with protease (Qiagen, Hilden, Germany) and then subjected to a serial of hybridization reactions with cocktail probes specific to the intended examined markers described above. Finally, we applied 4’,6-diamidino-2-phenylindole (DAPI) to stain the cell nucleus, and the cells were analyzed with an automated imaging fluorescent microscope (Carl ZeissMeditec AG, Jena, Germany). According to their morphology and biological biomarkers, CTCs were divided into epithelial, mesenchymal and hybrid phenotypes. Epithelial CTCs were tested by labeling epithelial markers, mesenchymal CTCs were detected with labeling mesenchymal markers, and hybrid CTCs were detected with labeling both epithelial and mesenchymal markers. Leukocytes were characterized as CD45+DAPI+ cells. A detailed procedure was described for reference.

### RNA *in situ* hybridization assay in CTCs

PD-L1, EpCAM, CK8, CK18, CK19, vimentin and twist gene expression levels from these different cell types were also detected by RNA in situ hybridization. We used sequences of CD45 (leukocyte biomarker), twist, Vimentin (the mesenchymal biomarkers) and EpCAM, CK8,CK18,CK19 (epithelial biomarkers), which have been published in Wu’s paper [20] and are shown in Table 3, to help distinguish epithelial, mesenchymal and hybrid phenotype CTCs.

**Table 3:** Multivariate analyses of progression-free survival and overall survival (n = 103).

	Progression-free Survival			Overall Survival		
Variables	HR	95%CI	P*	HR	95%CI	P*
Male vs. Female	0.188	0.024–1.472	0.111	2.242	0.158–31.88	0.551
Age	1.008	0.948–1.072	0.793	1.067	0.973–1.170	0.169
Squamous vs. Adenocarcinoma	0.922	0.837–1.046	0.356	1.018	0.982–1.159	0.626
Stage I vs. Stage IV	0.010	0.000–0.286	0.007	0.580	0.027–12.49	0.728
Stage II vs Stage IV	0.160	0.017–1.517	0.110	0.322	0.009–11.13	0.530
Stage III vs Stage IV	0.863	0.110–6.801	0.889	6.302	0.244–162.4	0.267
Preoperative CTC	12.48	0.258–604.8	0.202	0.265	0.013–5.243	0.383
Intraoperative CTC	1.013	0.135–7.595	0.990	3.141	0.285–34.65	0.350
Postoperative CTC	0.012	0.001–0.286	0.006	0.118	0.009–1.559	0.105
Pulmonary vein CTC	0.023	0.001–0.419	0.011	2.403	0.204–28.23	0.486
Neoadjuvant Chemotherapy	0.003	0.001–0.437	0.022	0.129	0.002–7.349	0.321
Neoadjuvant Radiotherapy	11.35	0.957–134.6	0.054	3.870	0.252–59.36	0.331
Adjuvant Chemotherapy	49.01	0.074–3255	0.240	17.89	0.344–804.3	0.735
Adjuvant Radiotherapy	1.512	0.284–8.046	0.628	9.115	0.573–145.1	0.118
Targeted Therapy	0.554	0.092–3.342	0.520	0.158	0.015–1.714	0.129
WBC-preoperative	0.816	0.335–1.404	0.723	0.922	0.205–1.592	0.845
WBC-postoperative	1.117	0.632–1.784	0.508	0.761	0.145–1.364	0.491
NLR-preoperative	0.547	0.324–0.895	0.739	0.672	0.437–0.901	0.318
NLR-postoperative	0.766	0.451–1.016	0.562	2.702	1.539–3.968	0.573
Immunotherapy	0.520	0.022–12.27	0.685	0.010	0.001–0.233	0.004

*Cox regression analysis was used to compare the statistical differences of various factors on survival

Preoperative CTC, Peripheral arterial blood CTC 7 days before surgery; Intraoperative CTC, Peripheral arterial blood CT during operation.

Preoperative CTC, Peripheral arterial blood CTC 7 days before surgery; Intraoperative CTC, Peripheral arterial blood CT during operation.

**Fig 1:**
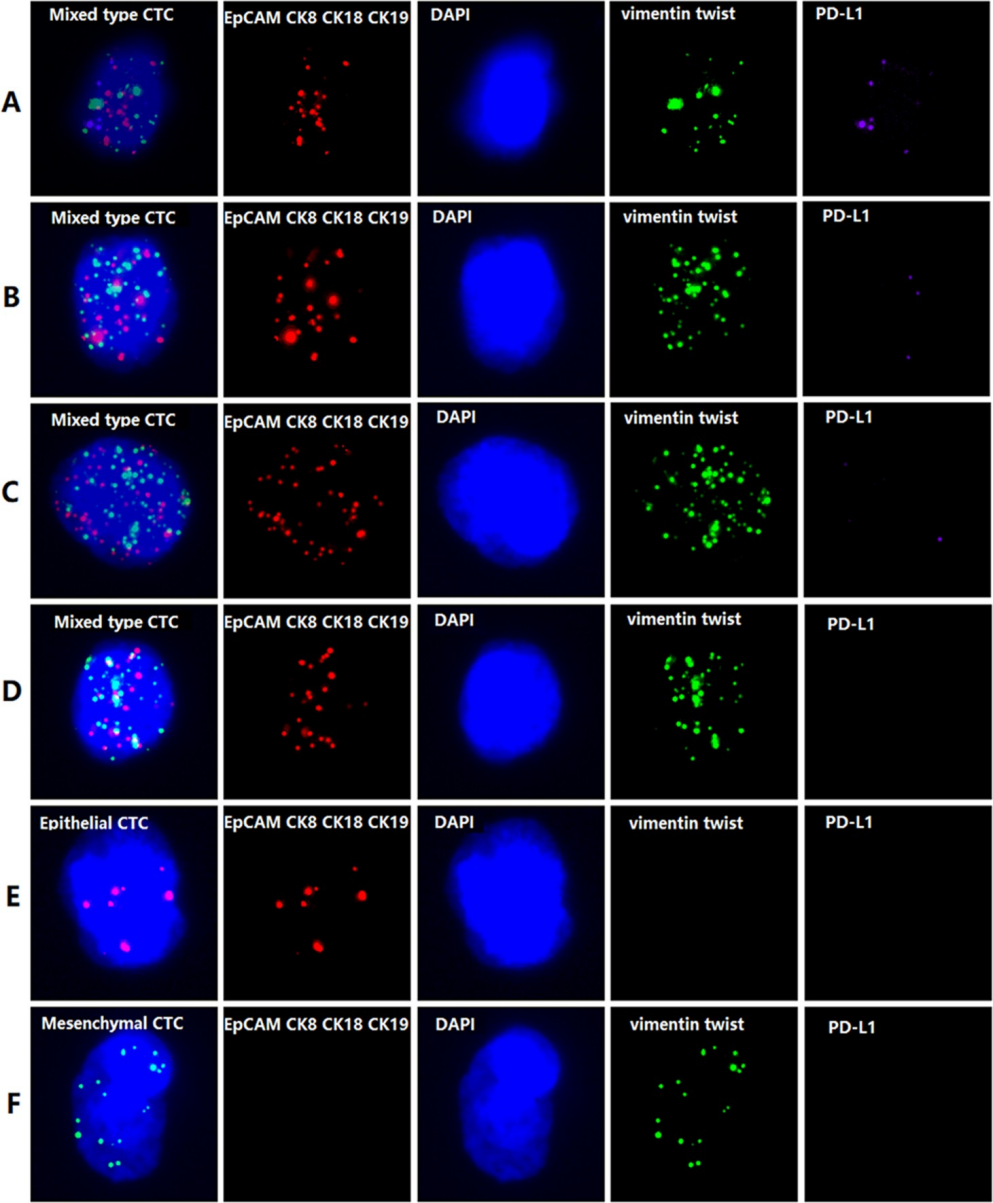
Classification and PD-L1 mRNA expression level in CTCs. Detection and classification of CTCs using multiple epithelial markers, including EpCAM, CK8/18/19 (red fluorescence) and mesenchymal markers such as Vimentin and Twist (green fluorescence). The PD-L1 mRNA expression level in CTCs was detected by RNA-ISH (purple fluorescence).

The expression level of PD-L1 mRNA in CTCs was also detected by the RNA-ISH assay (Fig 1). The capture probe was designed to capture specific PD-L1 mRNA, followed by conjugation to the branched DNA (bDNA) signal amplification probes to create a branched structure. Finally, the labeled probes conjugated with a fluorescent dye were hybridized to the bDNA sequence. The results were analyzed using a fluorescence microscope. PD-L1 expression was calculated bythe following method: if PD-L1 = 0 indicated no expression; 0<PD-L1<3 indicated low expression; PD-L1>3 indicated high expression; if PD-L1 >0 but <3, it indicated low expression; if PD-L1 was >3, it indicated high expression.

### PD-L1 mRNA expression analysis in NSCLC tumor tissues

**Fig 2:**
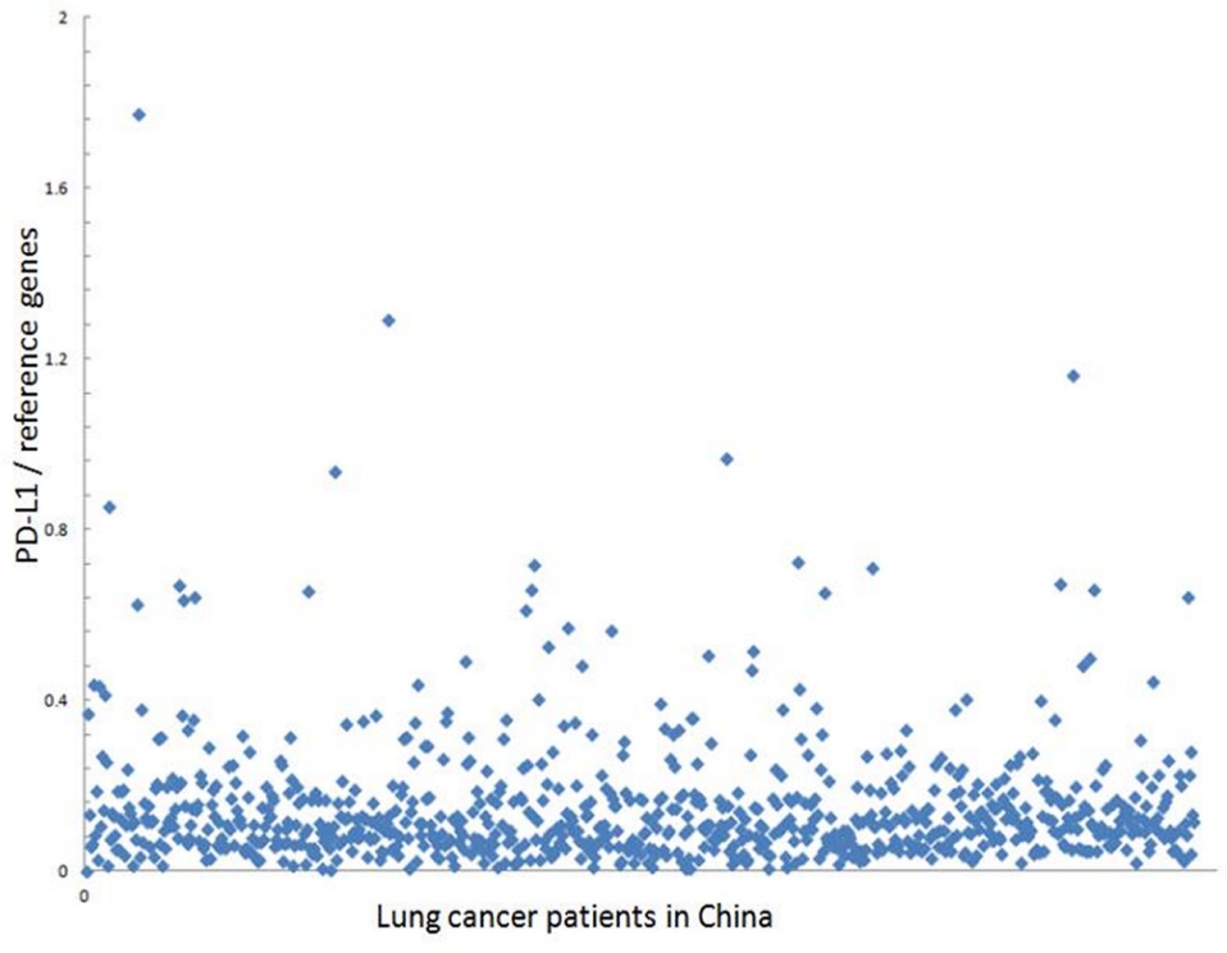
Expression level of PD-L1 in the patients. Expression level of PD-L1 and reference genes collected from lung cancer patients in China.

The mRNA expression of PD-L1 in tissue samples was assessed by the multiplex branched-DNA (bDNA) liquid-chip technology that was provided by Guangzhou SurExam Bio-Tech Co., Ltd., China.Firstly, the homogenate from the tissue sample was hybridized with target gene-specific probe sets and fluorescence capture beads. Next, the mixture was incubated with a series of bDNA signal amplification probes for signal amplification. Finally, the fluorescence value of each sample was collected using the Luminex 200 system. Three genes, the beta-2-microglobulin (B2M), TATA box-binding protein (TBP), and transferrin receptor (TFRC) genes, were used as reference genes. The raw data were normalized after adjustment, and the yielded values were the distribution of the patient’s gene expression among the whole population, representing the mRNA expression level in each patient. The mRNA expression of PD-L1 was collected from lung cancer patients in China, and the database was established by SurExam Bio-Tech Co. (Fig 2).The gene expression level was normally distributed. The detection data of the patients represents the proportion of patient with similar or identical test results in the database. According to the ratio of the expression level of the submitted sample in the database, the RNA levels were classified as low (L, the ratio was less than 25%), medium (M, the ratio was 25%-75%) and high (H, the ratio was over 75%) based on the quartile values.

### Expression of PD-L1 protein in NSCLC tumor tissues

One hundred three patients were selected for immunohistochemical (IHC) analysis. IHC staining was performed using the Dako Omnis system. The sections were deparaffinaged in xylene and rehydrated through a series of graded alcohols. Antigen retrieval was performed by incubating the sections. After antigen retrieval, the specimens were cooled for 30 minutes and then were incubated at 4°C for an hour using the PD-L1 primary antibody (1:25; Rat, Biospring, USA). After an hour of incubation with the primary antibody, the slides were incubated with a secondary reagent for 10 minutes and then were incubated with the polymer for 20 minutes (both provided by the Dako Omnis system). The slides were viewed under a microscope with an image capture system (OLYMPUS IX51). The expression rate of PD-L1 was determined by two experienced pathologists, and the average value was finally obtained. The method mentioned above is the same as it published literature[21].

All viable tumor cells were evaluated, with the presence of a minimum of 100 viable tumor cells required for adequate quantification pf PD-L1 expression. After correlated with a slide stained by hematoxylin and eosin, a trained pathologist in scoring PD-L1 expression scored any perceptible membranous staining (≥1+) of tumor cells and quantified the percentage of viable PD-L1 expressing tumor cells in the cytology or histology samples. Although multiple pathologists participated in scoring for the study, only 1 pathologist was assigned to each specimen. If an additional sample from a patient became available, its scoring was performed independently and without side-by-side comparison with its antecedent. Staining identified in necrotic cells or pulmonary alveolar macrophages was disregarded. PD-L1 expression was requantified by 2 additional pathologists in a randomly selected subset of cases (10 surgical resection, 10 cytology, and 10 histologic small biopsy specimens) to assess reproducibility. Additional pathologists reviewed the results and reached a consensus. Possible reasons for any discrepancy were noted.

### Statistical analysis

SPSS Statistics 19 software (IBM Deutschland GmbH, Germany) was used for statistical analysis. A *P*-value less than 0.05 was considered a statistically significant difference. GraphPad Prism 6.02 was used for image processing.

The survival curve of the OS and PFS of NSCLC patients were plotted by the Kaplan-Meier method after the log-rank test. OS was the time from surgery to death. PFS was the time from surgery to the time of diagnosis of local recurrence, distant metastasis or death, whichever occurred first.

The Cox regression model was used for multivariate analysis of all independent influence factors, including the CTC results and other factors, on OS and PFS. Kaplan-Meier curves were computed using GraphPad Prism 6.02.

T-test was used to compare and analyze continuous variable factors in this study. Chi-squared test was used to analyze the factors of categorical variables. Significance was indicated by the *P*-values of two-tailed tests <0.05.

## Results

### Patient characteristics and CTC detection

One hundred and three patients were included in this study. Fifty-four were female (52.4%), and fifty-nine were male (47.6%). Thirty-six squamous lung cancer patients (34.9%), sixty patients with adenocarcinoma lung cancer (58.3%), and seven patients with other histological types of NSCLC were included in this study.

Patients with stage I NSCLC accounted for the largest ratio (forty-eight). There were 19 patients with stage II NSCLC, 27 patients with stage III NSCLC and 9 patients with stage IV NSCLC. Stage IV NSCLC was determined when the tumor was found disseminated to the pleural membrane during surgery.

Three NSCLC patients accepted neoadjuvant chemotherapy, and only one NSCLC patient received neoadjuvant radiotherapy. Postoperative adjuvant chemotherapy and radiotherapy were performed in thirty-nine patients and twenty-nine patients separately. Seven patients received targeted therapy, and 2 patients received immunotherapy (Keytruda). The results are shown in Table 1.

In this study, CTCs in preoperative peripheral arterial blood were found in 89.3% patients (92/103); CTCs in pulmonary vein blood were found in 98.1% patients (101/103); CTCs in peripheral arterial blood during the operation were found in 94.2% patients (97/103). Only 76.6% patients (79/103) had CTCs in postoperative peripheral arterial blood.

### Comparison of CTCs at different stages

**Fig 3:**
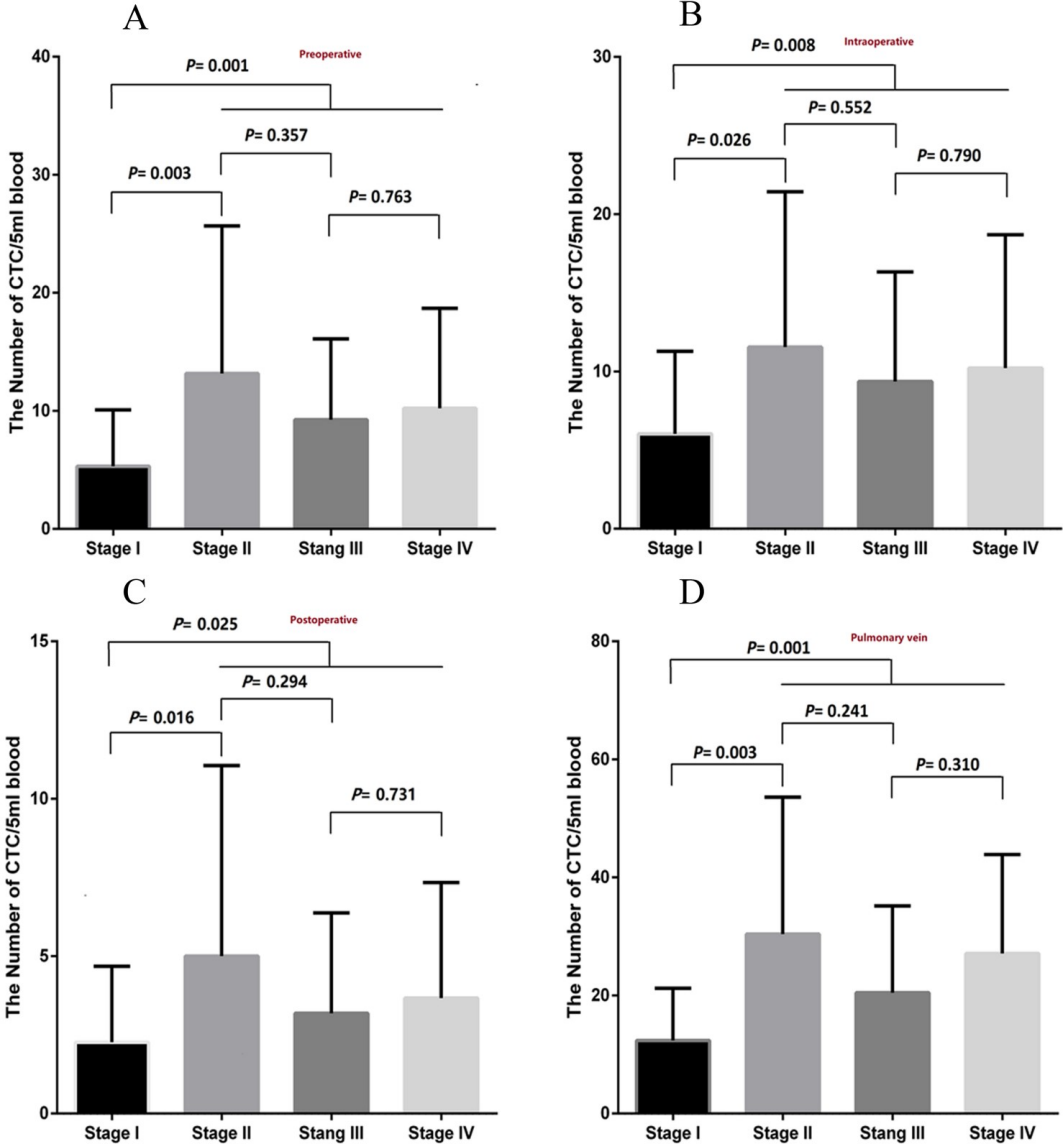
Comparison of CTCs at different stages. (A) Comparison of CTCs from peripheral arterial blood before surgery at different stages. (B) Comparison of CTCs from peripheral arterial blood during operation at different stages. (C) Comparison of the CTC number from peripheral arterial blood 1 hour after surgery at different stages. (D) Comparison of the CTC number from pulmonary venous blood extracted during surgery at different stages.

As for the CTC number in peripheral blood before surgery, during surgery and 1 hour after surgery, stage I patients had much lower number of CTCs than the stage II-IV patients (*P* = 0.001, *P* = 0.008, *P* = 0.025 respectively; Fig 3A–3C) However, no significant difference was found in the quantity among stage II, stage III and stage IV patients.

Furthermore, we compared the number of CTCs in pulmonary venous blood extracted during surgery, the number of CTCs in stage I patients was also significantly lower than that in stage II patients, and the difference was statistically significant (*P* = 0.001; Fig 3D). However, there was also no significant difference in the quantity among stage II, stage III and stage IV patients.

### Relationship between the tumor size and lymph node metastasis and CTC number

**Fig 4:**
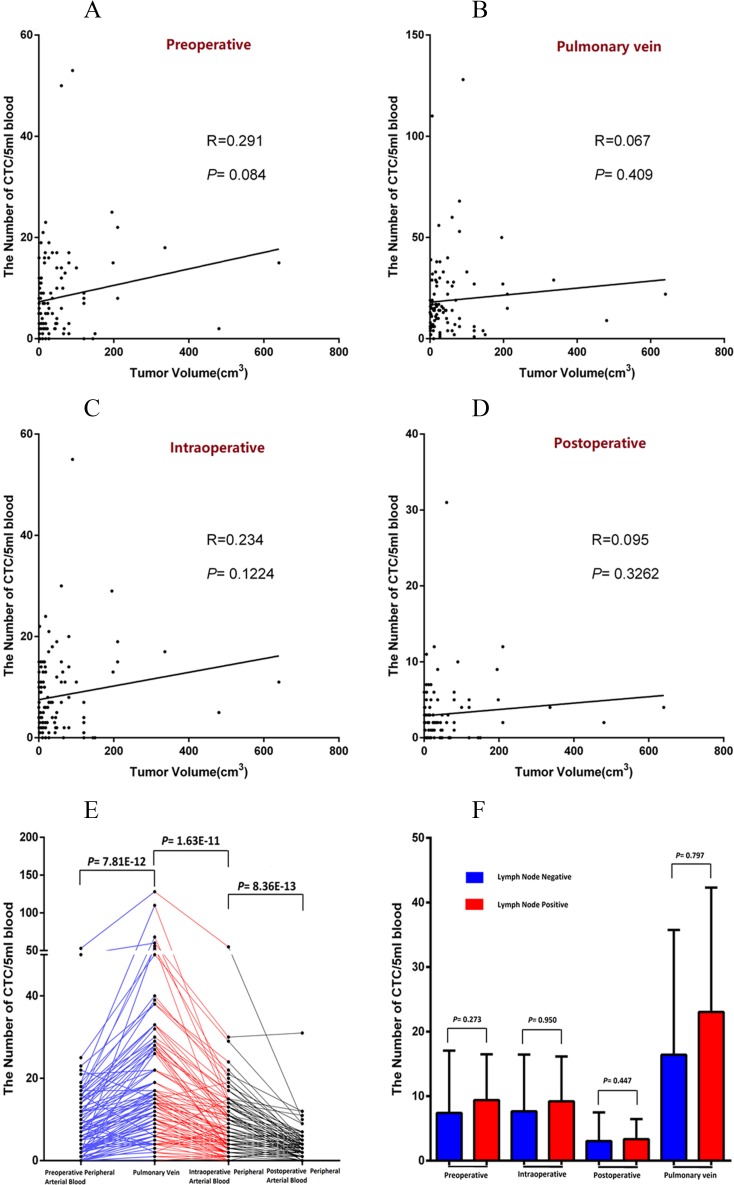
Relationship between the Tumor size and lymph node metastasis and CTC number. (A) in preoperative peripheral artery blood. (B) in the pulmonary vein blood extracted during surgery. (C) in peripheral arterial blood during surgery. (D) in peripheral blood 1 hour after surgery. (E) Comparison of the CTC number detected at the four time points above. (F) Comparison of the correlations between the CTCs in peripheral blood and pulmonary venous blood and mediastinal lymph node metastasis.

The tumor size of NSCLC patients may have a linear relationship with the number of CTCs in preoperative blood. However, it had no statistical significance (*P* = 0.084; R = 0.291; Fig 4A). The tumor size of the NSCLC patients showed no obvious linear relationship with the amount of CTC in the pulmonary vein blood extracted during surgery, and the linear relationship displayed no statistical significance (*P* = 0.409; R = 0.067; Fig 4B). The number of CTCs in peripheral arterial blood during surgery was detected and had no obvious linear relationship with the tumor size and no statistical significance (*P* = 0.1224; R = 0.234; Fig 4C). The same situation is reflected in the linear relationship between the CTC number in peripheral blood after surgery and tumor size (*P =* 0.3262; R = 0.095; Fig 4D). For the same patient, by comparing the number of CTCs detected at the four time points above, the number of CTCs in pulmonary venous blood was revealed to be the highest (the mean of 18.9), and the difference was statistically significant (P = 1.63E-11 and 7.81E-12; Fig 4E). However, 1 hour after surgery, the number of CTCs in the peripheral arterial blood of the patient was the lowest (the mean of 3.1), and the difference was statistically significant compared with that during surgery (P = 8.36E-13; Fig 4E).

Furthermore, we compared the correlation between the number of CTCs in peripheral blood and pulmonary venous blood and mediastinal lymph node metastasis. The results showed no significant correlation between the number of CTCs in peripheral arterial blood and number of CTCs in pulmonary venous blood and mediastinal lymph node metastasis (P> 0.05; Fig 4F).

### Univariate survival analysis

**Fig 5:**
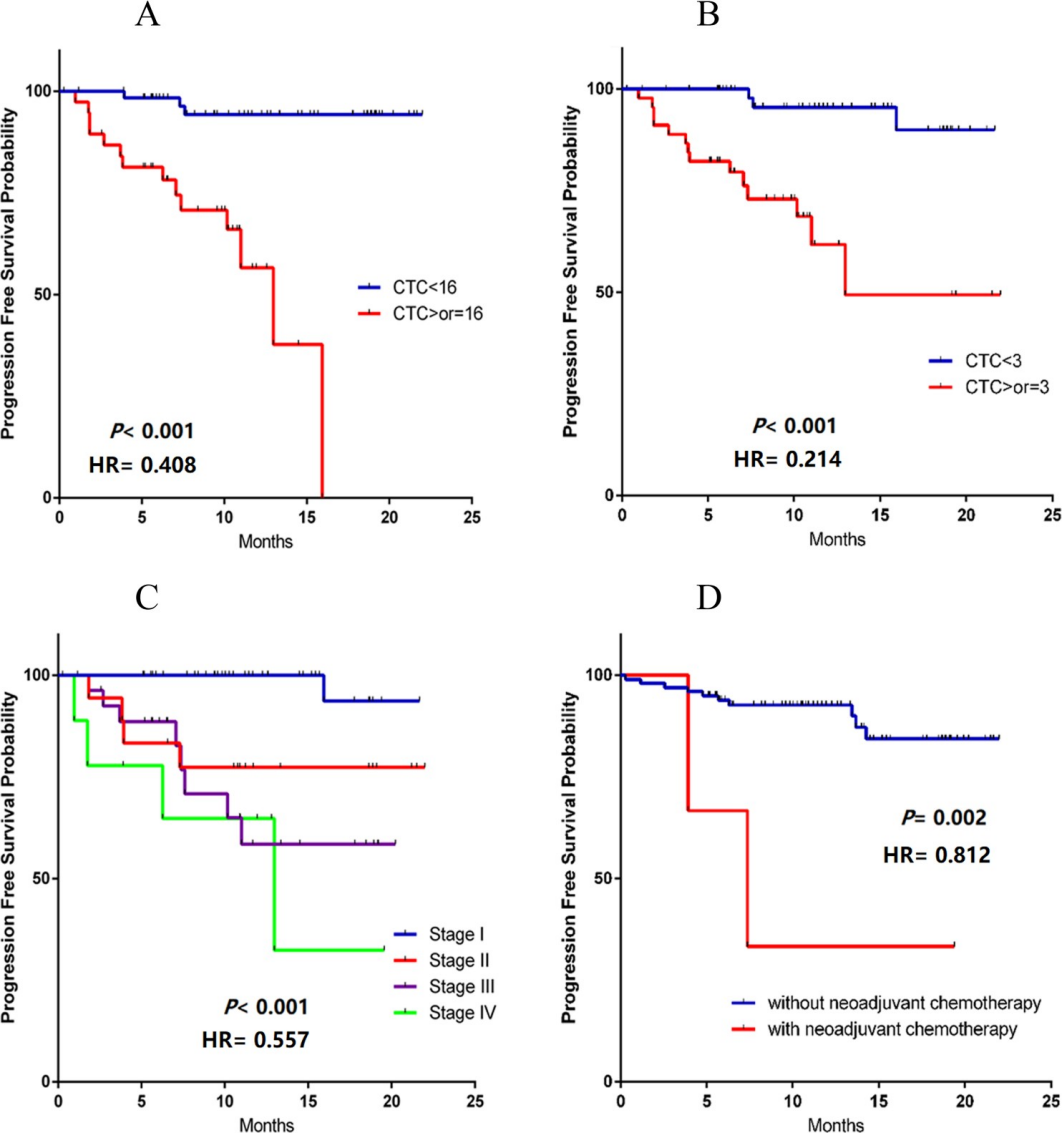
Univariate survival analysis. (A) The median postoperative progression-free survival (PFS) of the group with pulmonary vein CTCs≥16/5 ml patients (red) and group with pulmonary vein CTC<16/5 ml patients (blue). (B) PFS of the group with postoperative peripheral arterial blood CTC≥3/5 ml patients (red) and group with postoperative peripheral arterial blood CTC<3/5 ml patients (blue). PFS of the patients at different AJCC stages [Stage I (blue), Stage II (red), Stage III (purple), Stage IV(green)]. (D) PFS of patients with (red) or without (blue) neoadjuvant chemotherapy before surgery.

The median survival time of the whole group was 19.8 (CI: 18.7–21.0) months (range: 0–48 months). The median postoperative progression-free survival (PFS) was 18.6 (CI: 17.2–20.1) months in the whole group (range: 0–48 months), 11.1 (CI: 9.1–13.2) months (range: 0–48 months) in the group with pulmonary vein CTCs≥16/5 ml patients, and 21.2 (CI: 20.1–22.1) months (range: 0–48 months) in the group with pulmonary vein CTCs<16/5 ml patients (*P*< 0.001, Fig 5A).

The median postoperative progression-free survival (PFS) rates were 14.8 (CI: 11.6–17.9) months (range: 0–48 months) in the group with postoperative peripheral arterial blood CTCs≥3/5 ml patients and 20.7 (CI: 19.7–21.6) months (range: 0–48 months) in the group with postoperative peripheral arterial blood CTCs<3/5 ml patients (*P*< 0.001; Fig 5B).

The postoperative progression-free survival period of the patients was affected by the AJCC stage. The progression-free survival period of stage I patients was significantly the longest, while that of stage IV patients was the shortest (*P*< 0.001; Fig 5C). Patients who received neoadjuvant chemotherapy before surgery (n = 3) had a shorter survival period without progression after surgery (*P* = 0.002; Fig 5D).

### Multivariate survival analysis

All the clinical data of the patient including pulmonary venous blood CTC (HR: 0.023; CI: 0.001–0.049; *P* = 0.011; Table 3), postoperative peripheral arterial blood CTC (HR: 0.012; CI: 0.001–0.286; *P* = 0.006; Table 3) and AJCC stage were independent factors for progression-free survival.

Patients receiving postoperative immunotherapy (only 2 patients were stage IV) had a shorter overall survival period (HR: 0.010; CI: 0.001–0.233; *P* = 0.004; Table 3).

### Expression of PD-L1 mRNA and PD-L1 protein in NSCLC tissues

**Fig 6:**
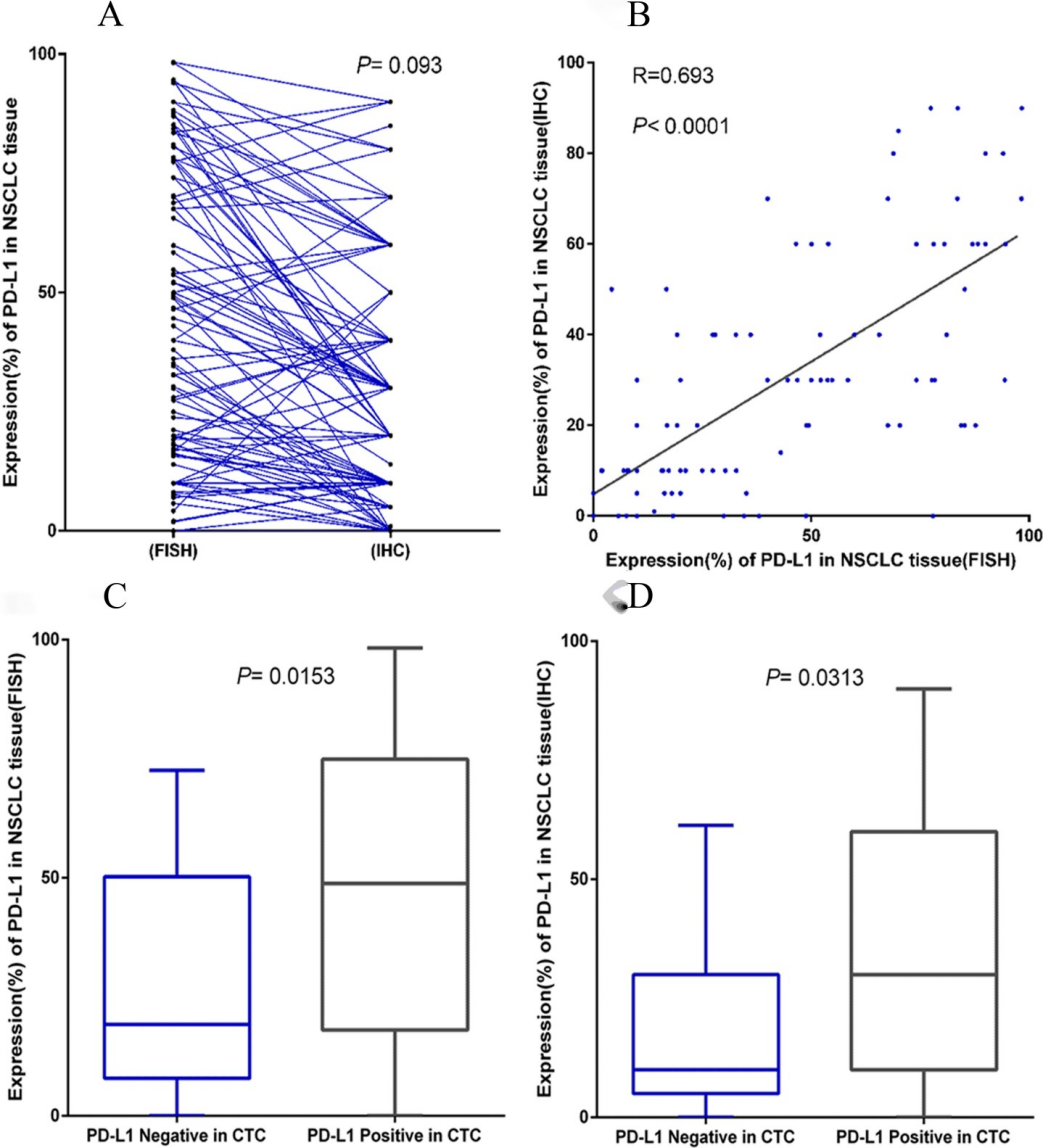
Expression of PD-L1 mRNA and PD-L1 protein in NSCLC tissues. (A) Comparison of PD-L1 expression in NSCLC tissues by FISH and IHC. (B) Relationship between PD-L1 expression in NSCLC tissues by IHC and FISH. (C) Comparison of PD-L1 expression in NSCLC tissues by FISH in PD-L1-negative and -positive CTCs. (D) Comparison of PD-L1 expression in NSCLC tissues by IHC in PD-L1-negative and -positive CTCs.

In this study, PD-L1 expression in NSCLC tissues was detected by comparing FISH and IHC. The results of the two methods were basically the same in tumor tissue of NSCLC patients (Fig 6A) and the difference was not statistically significant (*P* = 0.093).The results of the two methods were analysed by linear regression, and the linear relationship between IHC and FISH was found to be statistically significant (*P*< 0.0001; R = 0.693; Fig 6B).

### Expression of PD-L1 in pulmonary venous CTCs

PD-L1 expression in pulmonary venous CTCs was detected in all the patients. Fifty-two (50.5%) patients had positive PD-L1 expression in CTCs. In NSCLC tissues of patients with positive PD-L1 expression in CTCs, the mean value of PD-L1 expression detected by FISH was 47%, significantly higher than that of the negative PD-L1 expression (mean value of 35.3%) group in CTCs (*P* = 0.0153; Fig 6C). Similarly, in the NSCLC tissues of patients with positive PD-L1 expression in CTCs, the mean value of PD-L1 expression detected by IHC was 33.7%, significantly higher than that of the negative PD-L1 expression (mean value of 24.5%) group in CTCs (*P* = 0.0313; Fig 6D).

## Discussion

The pulmonary vein is the nearest drain vessel to lung cancer, so it is an important route for the metastasis of tumor cells to the peripheral circulation in NSCLC patients. Even without specific metastatic lesions, NSCLC tumor cells could pass through the pulmonary vein into peripheral blood from the primary lesion [22]. Okumura et al. [23] found that CTCs in the pulmonary vein were significantly higher than those in peripheral blood. In this study, CTC tests were performed at four time points in every NSCLC patient who received surgery. The four time points were preoperative, intraoperative, postoperative and intraoperative pulmonary veins. The number of CTCs in pulmonary veins was significantly higher than that in the other groups (Fig 4E). These results suggested that CTC was easier to find in the pulmonary vein because the blood of entire tumor needed to flow back through a single pulmonary vein. As Reddy's research has confirmed, the method of detecting CTCs in the pulmonary veins is superior in the study of early-stage NSCLC patients [24]. In this study, we found that the number of CTCs in the blood of patients with advanced stage was significantly higher than that in patients with early-stage NSCLC, regardless of the time point (*P*< 0.05; Fig 3A–3D).

The determinants of the stage of NSCLC include tumor size, lymph node metastasis and distant metastasis. Clinically, NSCLC patients are often found with small primary tumors (<2 cm) but multiple metastatic lesions throughout the body. There is still a 25–50% recurrence rate in patients with NSCLC who have received surgical treatment [17, 25, 26]. These findings suggested that tumor cells are metastasized from primary lesions through the pulmonary vein in patients with early NSCLC. As found in this study, no strict linear relationship existed between the tumor size and CTC number in the patient's blood (*P*> 0.05; Fig 4A–4D).

Lymph node and blood are metastatic pathways of NSCLC. In this study, we found that the number of CTCs in the patient's blood was not associated with lymph node metastasis (*P*> 0.05; Fig 4F). These results suggested that the blood spread to blood and metastasis to lymph nodes are independent processes with different mechanisms.

In general, most of the patients with early NSCLC do not require adjuvant treatment after surgery. However, in a small subset of these patients, tumor recurrence or metastasis occurs. Thus it is critical to evaluate the prognosis of early NSCLC patients. The relationship between the CTC number and prognostic value has been demonstrated in breast and colon cancer [27, 28]. Many studies have found that CTC can be a good prognostic marker for NSCLC patients [29]. However, the cutoff values used for prognosis were also different due to different CTC detection platforms [30, 31]. In this study, multivariate survival analysis found that UICC staging, CTC number in pulmonary venous blood and CTC number in postoperative peripheral arterial blood were independent risk factors for patient prognosis (Table 3, Fig 5C). The postoperative progression-free survival was significantly shorter in patients with more than 15/5 ml CTCs in pulmonary veins (***P*<0.001;** Fig 5A). The postoperative progression-free survival was significantly shorter in patients with CTCs greater than 2/5 ml in postoperative peripheral arterial blood (***P*<0.001;** Fig 5B). The cut-off value of CTCs number in pulmonary veins and postoperative peripheral arterial blood are determined with the median. In this study, the amount of CTCs was not significantly related to the postoperative overall survival due to various factors such as postoperative radiotherapy and chemotherapy. Two patients with stage IV who received immunotherapy (Keytruda) before surgery had a shorter survival. The two patients had stage IIIB disease before surgery, and pleural metastasis was found during surgery; the final stage after surgery was stage IV.

The method of pulmonary vein blood drawing in the thoracotomy surgery (Open Chest) is simple relatively. When the proximal end of the pulmonary vein is ligated, blood can be drawn at the distal end of the pulmonary vein. In less-invasive surgical approaches (VATS), the pulmonary venous blood drawing procedure becomes relatively difficult but can still be done. We recommend that the proximal pulmonary vein be ligated with an intraventricular knotter and a needle with an extension tube be placed in the chest cavity to draw blood from the distal pulmonary vein. After this procedure, the intramural incision suture device can be used again to cut the pulmonary vein.

Some scholars have already found that WBC[32] and neutrophil-to-lymphocyte ratio[33] can be immune-related markers of prognosis of NSCLC. In this study, WBC results and neutrophil-to-lymphocyte ratios were measured preoperatively and at 1 hour after surgery. Then We incorporated the above two indicators into multivariate cox regression analysis and found that neither of them were independent risk factors for prognosis. It is possible that WBC and neutrophil-to-lymphocyte ratio are not the most important prognosis factors. Many factors can affect the amount of WBC in patients, such as smoking, inflammation, stress state, viral infection and so on.

The detection of CTCs in the blood may indicate important information on tumor progression, tumor typing and treatment selection [34]. The PDX model constructed by amplification of CTCs in vitro for drug screening and drug resistance mechanism will probably eradicate tumor metastasis [35]. In nonsurgical NSCLC patients, it is sometimes difficult to obtain biopsy samples. In advanced NSCLC patients, re-biopsy is often required after the disease progresses, making it more difficult to obtain tissue samples. The detection of CTCs can reflect real-time tumor progression in patients [36], and the detection of molecular markers in CTCs can provide an important basis for the selection of therapeutic methods [37], especially EGFR detection in CTC [38]. The tumor-expressed programmed death ligand (PD-L1) will downregulate T-cell activation and promote immune escape when binding with programmed death 1 (PD-1) protein expressed on the T-cell surface [39]. Nivolumab, the first PD-L1 inhibitor, was approved for the treatment of NSCLC in 2015 [40]. Studies [41, 42] have confirmed that the expression of PD-L1 in tumors is significantly correlated with the treatment response of nivolumab. In general, Immunohistochemistry methods were applied to detect PD-L1 in tumors[43]. Previous studies have shown that gene target detection in CTCs can guide targeted therapies. As a good indicator of immunotherapy, can we detect PD-L1 in CTCs? Thus far, only a few studies have investigated PD-L1 expression in the CTCs of NSCLC [44, 45]. In our study, the expression of the PD-L1 gene was detected in CTCs from the pulmonary vein. The method of drawing blood from pulmonary veins may increase the detection efficiency of PD-L1 in CTCs. The expression ratioof PD-L1 in tumor tissues of the PD-L1-positive group in CTCs was significantly higher than that of the PD-L1-negative group (*P* <0.05, Fig 6C and 6D). Obviously, patients who can be detected PD-L1 positive CTC in the blood may be more suitable for immunotherapy.
